# The experience of women with recent gestational diabetes during the COVID-19 lockdown: a qualitative study from Denmark

**DOI:** 10.1186/s12884-022-04424-5

**Published:** 2022-01-29

**Authors:** Nanna Husted Jensen, Karoline Kragelund Nielsen, Inger Katrine Dahl-Petersen, Helle Terkildsen Maindal

**Affiliations:** 1 Department of Public Health, Aarhus University, Aarhus, Denmark; 2 Health Promotion Research, Steno Diabetes Centre Copenhagen, Herlev, Denmark

**Keywords:** Gestational diabetes mellitus, COVID-19, Lockdown, Coronavirus, Health care delivery, Mental health, Diabetes prevention, Motherhood adaptation, Infant’s health

## Abstract

**Background:**

Following COVID-19 and the lockdowns, maternity care and support for women after delivery have been temporary restructured. Studies show that COVID-19 adversely impacts pregnant and peripartum women in the general population, but experiences among women in the first year after delivery/in the wider postpartum period remain unexplored. Moreover, experiences among women with recent gestational diabetes mellitus (GDM) are lacking; though it is a group with a potential high need for support after delivery. The aim of our study was to investigate (i) how women with recent GDM experienced COVID-19 and the first lockdown in Denmark, and (ii) the women’s risk perception and health literacy in terms of interaction with the healthcare system in relation to COVID-19.

**Methods:**

We performed a qualitative study among 11 women with recent GDM (infants aged 2-11 months old). Semi-structured interviews were conducted in April-May 2020 by telephone or Skype for Business, when Denmark was under lockdown. We analysed data using a thematic qualitative content analysis.

**Results:**

Three themes emerged: *i) Everyday life and family well-being*, ii) *Worries about COVID-19* and *iii) Health literacy: Health information and access to healthcare*. The women were generally not worried about their own or their infant’s risk of COVID-19. The lockdown had a negative impact on everyday life e.g. routines, loneliness, breastfeeding uncertainties and worries for the infant’s social well-being; but better family dynamics were also described. It was challenging to maintain healthy behaviours and thus the women described worries for the risk of type 2 diabetes and GDM in subsequent pregnancies. The women missed peer support and face-to-face visits from health visitors and found it difficult to navigate the restructured care with online/telephone set-ups.

**Conclusions:**

COVID-19 and the lockdown affected everyday life among women with recent GDM both positively and negatively. Our findings suggest a need for care that are responsive to psychological and social aspects of health throughout the COVID-19 pandemic and support to limit worries about adaptation to motherhood and the infant’s social well-being. Communication focusing on the importance and relevance of contacting healthcare providers should also be strengthened.

## Introduction

In January 2020, the World Health Organization declared the coronavirus (SARS-CoV-2) outbreak and its associated disease (COVID-19) a public health emergency of international concern [1, 2]. Multiple countries have since implemented diverse strategies to limit coronavirus transmission [3]. On 27 February 2020, the first Danish citizen tested positive for COVID-19 [4], and on 11 March, Denmark imposed the first lockdown which involved the closure of public places, schools, day care institutions, social distancing and restriction of gatherings. Moreover, healthcare services were restructured during the lockdown e.g. by postponing none-immediate treatments and changes to how care was delivered. Care for families with an infant (0-1 years old) was also restructured.

Gestational diabetes mellitus (GDM) is one of the most common pregnancy complications, affecting approximately one in seven births globally [5]. In 2018, 4.7% of all births in Denmark were affected by GDM [6]. In the short term, GDM is associated with adverse maternal and neonatal outcomes, such as pre-eclampsia, foetal macrosomia and caesarean section [7]. In the long term, women who have been diagnosed with GDM have a up to 10-fold increased risk of type 2 diabetes mellitus (T2DM) compared to women with a normoglycemic pregnancy [8, 9]. Offspring exposed to GDM in-utero also have an increased risk of developing T2DM and prediabetes in later life as compared to offspring of women with a normoglycemic pregnancy [10]. In addition, women with recent GDM also have higher rates of postpartum depression compared to women without GDM during pregnancy [11, 12]. Hence, this group needs additional support after delivery to ensure good health and well-being [8, 13].

Studies have found that pregnant women and perinatal women in general have experienced challenges during COVID-19 [14–18]. Poorer mental health and difficulties in engaging with stress-coping behaviours have been identified among these women during times with COVID-19 restrictions and lockdowns [15, 19]. A study from Italy found that pandemic-related maternal anxiety may also have increased parenting stress and reduced maternal boding [20], and a number of studies report unfavourable experiences of the healthcare services during pregnancy and postpartum [17, 21]. However, to our knowledge there is little existing evidence on the experience of COVID-19 in women in the wider period after delivery, though transition and adaptation to motherhood during this period can be highly challenging and have potential adverse consequences for both the mother and the child [22–24]. These challenges may be pronounced among women who have had a complicated pregnancy e.g. women with GDM during times with COVID-19. The aim of our study was to investigate (i) how women with recent GDM experienced COVID-19 and the first lockdown in Denmark, and (ii) the women’s risk perception and health literacy in terms of interaction with the healthcare system in relation to COVID-19.

## Methods

### Design

This is a qualitative study based on semi-structured interviews with women with recent GDM during the COVID-19 lockdown in Denmark. We choose an interview approach to establish an in-depth understanding of the situation and gain insights into participant’s experiences, feelings and beliefs [25].

### Participants

We interviewed 11 women from three cities in Denmark (Aarhus, Odense and Copenhagen). All of the women had been diagnosed with GDM in their recent pregnancy. The women were recruited from a research project (the Face-it study) aiming to prevent T2DM among women with GDM through a health promotion intervention [26]. Approx. 120 women were recruited to the Face-it study when this qualitative study was initiated [26]. In April 2020, we invited 45 of the women in the Face-it study by e-mail to participate in our interview; 13 responded positively and 8 declined to participate. We received no response from the remaining women. It was not possible to establish contact with 2 of the 13 women subsequently. Eligibility criteria for participants in this study were consistent with the Face-it study [26]. However, we recruited women to maximise geographical representation across the three Danish cities and parity status; thus, the women represented both first-time mothers and women with more than one child. No systematic analysis was performed to assess potential differences between invited non-participants and the women who agreed to participate in the interviews. We continued to recruit and interview women until data saturation was reached [27].

### Setting

We collected our data from 24 April to 26 May 2020, i.e. during the first months of the COVID-19 pandemic in Denmark. The first lockdown in Denmark started on 11 March 2020. There were some initial re-openings from mid-April 2020. Thus, our study was undertaken when some lockdown initiatives were still ongoing.

In Denmark, there is free and equal access to most healthcare services. After delivery, families receive care from health visitors (nurses educated in child and maternal healthcare) until the infant is approximately 10 months old. In normal circumstances, health visitors focus on infant’s well-being, family functioning and provide supervision on e.g. breastfeeding and diets. The services are usually offered during face-to-face visits in the family’s home. In addition, after delivery women are usually invited to take part in a “mothers’ group”, which aims to facilitate peer support.

During the March 2020 lockdown in Denmark, maternity care and standard supportive care after delivery for women were changed. Many of the regular home visits were postponed or cancelled and families were advised to establish contact by telephone if they felt they needed support and supervision. In general, face-to-face contacts were prioritised for more acute concerns. Moreover, mothers’ groups (peer support) were paused or cancelled.

### Data collection

We developed a semi-structured interview guide with open-ended questions that focussed on women’s experiences during COVID-19 and the lockdown. The interview guide was qualified by a health visitor, who has regular contact with families in the first year after delivery. Some questions were based on theoretical terms, i.e. health literacy and risk perception. Health literacy is defined as an individual’s ability to access, understand, appraise and use health information and services to make decisions about their health [28]. We included health literacy questions to learn about women’s access to and use of health information and healthcare services during COVID-19. Examples of interview questions concerning health literacy were *“Have you been in contact with your health visitor, general practitioner or similar during the COVID-19 lockdown? If yes, can you give an example? If no, why has it not been necessary for you to contact these?”* and *“How did you experience the information about coronavirus delivered by the media, health authorities etc.?”.* For the risk perception questions we wanted to understand how the women experienced their individual risk in relation to COVID-19 and whether they took any precautions to limit their own risk of contracting coronavirus. Examples of risk perception related questions were *“Please tell me about your potential concerns for your own and your child’s risk during the COVID-19 pandemic?”, “Do you think your recent GDM diagnosis is associated with your concerns about your own risk of contracting coronavirus? If yes, in which direction, If no, why not?”.* The remaining questions were of an explorative nature to examine individual experiences of COVID-19 and the lockdown. All interviews were performed by a health promotion researcher (NHJ) on the telephone or using Skype for Business as online media depending on the participant’s preference. Each interview was recorded on a voice recorder. The interviews lasted between 28 and 59 min (mean = 39 min).

### Analysis

The interviews were transcribed verbatim by NHJ and one assistant. We used qualitative content analysis to organise and interpret data across major themes that arose from our data [29, 30]. We coded the transcripts by applying categories to each sequence of text (‘meaning units’ of text) following a largely inductive approach to condense the material [29]. The meaning units were categorised into different themes to gain an overview of the data. Table 1 demonstrates the analysis process. The analysis was performed by NHJ, who is experienced in conducting qualitative research and within the research field of health promotion among women with recent GDM and child and maternal healthcare. KKN assisted the analytical process for categorising the meaning units into themes, and we applied peer triangulation [31] meaning that KKN, IKDP and HTM also reassessed final interpretations of the data condensation. In order to achieve trustworthiness and evaluate the study procedures used to generate findings we applied credibility, dependability and transferability as concepts [29]. Credibility concerns questions related to the focus of the study, selection of context and participation and approach for gathering data. Hence, we strived to recruit women according to the before mentioned criteria to obtain variation in data. Moreover, efforts were made to establish a safe environment for sharing of experiences and feelings even though interviews were performed by telephone and skype.

**Table 1:** Examples of the analytical process; meaning units and their associated condensed sub-category, category and theme

Meaning unit (text sequence)	Sub-category	Category	Theme
*” Right now, I would not use my general practitioner in order to avoid spamming them with something that does not need to be addressed, if one can get information from elsewhere”*	Limited contact with healthcare providers to avoid unnecessary contacts that are not urgent	Contact with healthcare providers	*Health Literacy: Health information and access to health care*
*“So being able to compare [Infant’s progression] and have these typical conversations in your mothers’ group, for example your infant’s progression and how you introduce solid food and all these everyday practical questions. They are more difficult to address elsewhere, and then you are left on your own”*	Lack of peer-to-peer support introduces worries for managing infant’s needs and a feeling of being left alone without support	Support from peers	*Everyday life and family well-being*

## Results

As shown in Table 2, the participants’ age ranged from 28 to 38 years old and six of them were first time mothers whereas the rest had two or more children. All women were living with a partner and had a high educational level (high school and vocational school or higher). Two of the women were living in Copenhagen, five in Aarhus and the remaining four women were living in Odense. Infant’s age ranged from 2 to 11 months.

**Table 2:** Participants’ characteristics at the time of the interviews

	Age (years)	Number of children	On maternity leave/employed^a^	Education level	Infant’s age (months)	Cohabitation status
Woman no. 1	35	1	Maternity leave	University level	7.5	Living with a partner
Woman no. 2	35	2	Employed	High School and Vocational School	11	
Woman no. 3	37	1	Maternity leave	University level	8	
Woman no. 4	35	1	Maternity leave	University level	9.5	
Woman no. 5	35	1	Maternity leave	University level	6	
Woman no. 6	34	2	Maternity leave	University level	7	
Woman no. 7	31	1	Maternity leave	University level	2	
Woman no. 8	28	2	Maternity leave	High School and Vocational School	7.5	
Woman no. 9	38	2	Maternity leave	University level	9	
Woman no. 10	33	3	Employed	University level	10	
Woman no. 11	31	1	Maternity leave	High School and Vocational School	9.5	

^a^All women who were on maternity leave were either employed or enrolled in educational programmes prior to this leave

Three consistent themes emerged from our analysis; i) *Everyday life and family well-being,* ii) *Worries about COVID-19,* and iii) Health literacy: *Health information and access to healthcare.*

i) *Everyday life and family well-being*

The lockdown influenced various aspects of everyday life among the women. The women perceived these changes as both positive and negative. Some experienced a better family dynamic and a closer partner-infant relationship. In general, they appreciated having their partner more at home. Of other positive experiences, a first-time mother described her infant being more comfortable and relaxed because of more stable routines in relation to sleep and meals during the lockdown.

*“Before, we went out for so many things... so it was difficult to establish these regular routines for sleep and food etc. So, for example, in relation to her naps during the day; instead of us having to go with her in the pram, we are now able to put her to bed right away because she is used to sleep at the same time. Now there is nothing to disturb these routines”* [Woman no. 1]

In contrast, most of the women also found the lockdown difficult to manage. The women described feelings of loneliness and related this to changed circumstances during the lockdown.

*“I can’t really tell why it has been so difficult because I have gone on maternity leave before this [COVID-19]. But you know, being alone at home with two children when you actually do not have any time off for relief and at the same time you are not able to do anything with them [Because of the lockdown]. I also find it really hard to acknowledge that I have to spend the rest of my maternity leave like this”* [Woman no. 2]

The women highlighted the lack of peer support from mothers’ groups and the home visits from the health visitors as factors which made it difficult to adapt to motherhood during the lockdown. Under normal circumstances without COVID-19, the women described their “informal” talks with the health visitors during home visits as an opportunity to share worries about motherhood insecurities as well as the infant’s social progression etc. However, the women experienced this informal interaction as not being available when health visitor care was restructured and moved to telephone/online set-ups. While a lack of confidence in the motherhood role was also to some extent present before COVID-19, the women reported that their worries in relation to this intensified during lockdown and they felt left alone in how to handle them.

*“So it's such a strange feeling that now I think there are many things that I have confidence in. But I still cannot help but think if there is something I have overlooked or something I have done wrong…. For now it's so long ago that a health visitor has seen X [Infant’s name] and it's so long since I've seen my mothers’ group and it's been a long time since we've received any guidance” [woman no. 3]*

In relation to motherhood insecurity, some women worried for their infant’s social well-being and whether the minimal level of social interaction during lockdown would affect their child’s social progression negatively. Moreover, the women described reduced interaction with their social network during lockdown and feared that the limited contact would impact the infant’s ability to relate socially.

Furthermore, the lockdown had both positive and negative impacts on health behaviours such as physical activity and dietary behaviours. Since their partners were more at home, a few of the women found more time to do physical activity and cook meals. However, the majority described themselves as less physically active. Some women felt less motivated to change their behaviour e.g. physical activity, despite their understanding and acknowledgement of the importance of initiating healthy behaviours after delivery. The lack of motivation was influenced by tiredness, having children at home full time and lack of peer and social support. Although, these barriers were part of everyday life before COVID-19, they seemed to be intensified during lockdown.

“*It has clearly gone downhill in terms of exercise. For me specifically also getting out for walks. The routine changes because all of a sudden we have all the children at home at the same time, so you have to prioritise time differently”* [Woman no. 10]

In terms of being physically active, the lockdown introduced new barriers, such as not being able to attend fitness and postpartum training and altered routines due to restrictions e.g. not walking to school or day-care institutions to pick up children, suggesting a less physically active everyday life for many of the women. Overall it became more challenging for the women to prioritise physical activity during the lockdown.

Because of the difficulties in establishing healthy behaviours, some women reported increased worries about their T2DM risk and especially the risk of reoccurrence of GDM in subsequent pregnancies compared to times before the lockdown. Addressing this worry was particularly difficult when usual health visitor support as well as peer-to-peer support were lacking.

*“I might soon have a new baby. However, I feel that my good initiatives are paused, you know, my progression during COVID-19, I am worried how the future will look like if I am not doing something about it now”* [Woman no. 4]

In general, the participants felt obliged to prioritise their children’s needs over their own and they strived to normalise everyday routines as best as possible.

ii) *Worries about COVID-19*

In general, the women did not consider themselves at high risk of contracting coronavirus or experiencing adverse health outcomes due to COVID-19. A high level of trust in the health authorities and the healthcare system minimised the women’s worries about COVID-19. As information in general showed that there was not an increased risk for infants, the women were not worried about their children contracting coronavirus. Moreover, none of the women considered themselves to be at particular risk because of their recent GDM diagnosis.

Rather, worries related to contracting coronavirus were focused on its potential impact on everyday routines e.g. how to establish an isolation strategy in the family setting and how to take care of the infant during times with minimised possibilities for help among relatives due to social restrictions. A first-time mother with a 9 month old infant linked her worries to her motherhood insecurity and the changed circumstances during lockdown.

*“If I get it [COVID-19] and if I get sick, who will then take care of my daughter? Yes, my husband is here too, but if we all get sick? It is those kinds of thoughts and if it is just me who get sick, I do not know how I will deal with it”* [Woman no. 11]

Furthermore, some of the women were concerned whether COVID-19 would negatively affect breastfeeding and they felt uncertain about handling potential lactation difficulties if they contracted coronavirus. Moreover, women described that there had been no focus on potential lactation issues from health visitors during times with lockdown; especially in relation to communicating how to maintain breastfeeding in case of COVID-19 infection or whether it was not safe to breastfeed during a COVID-19 disease course. These uncertainties revealed new types of breastfeeding uncertainties among the women and they requested more focus on this from the health visitors. A woman with a 2 month old infant also wanted health information specifically related to infants and COVID-19 and found this information difficult to access.

*“So, his [the infant] risk - that is the most important thing. And the second thing that has worried me was what if I get infected? Not because I have been afraid of becoming sick but in terms of breastfeeding. How will it influence breastfeeding if I suddenly get a fever for five days or a cold or whatever it is that comes along when you get corona?”* [Woman no. 7]

Despite worries about the derived consequences from COVID-19, the women reported the importance of face-to-face social contact with their network. They found it important to expose their infants to others to keep their social well-being and progression on track.

iii) *Health literacy: Health information and access to healthcare*

Even though the women experienced intensified worries during the lockdown and felt a need to consult their health visitor or general practitioner during the lockdown, they described limited contact with them and health literacy difficulties. A first-time mother described she did not want to burden the health visitor or the general practitioner, because she expected them to be busy with COVID-19 related tasks. Others did not expect their health visitors to be able to answer questions regarding COVID-19, e.g. potential risks for infants in case of contracting coronavirus.

*“Right now, I would not use my general practitioner in order to avoid spamming them with something that does not need to be addressed, if one can get information from elsewhere.”* [Woman no. 9]

Instead, the women searched for information online and among their social network. Nevertheless, some women still requested more support and information. A first-time mother described wanting more support during the lockdown e.g. on how to tackle children’s needs. She felt that the available public information and support were prioritised for other groups, such as pregnant women, at the expense of herself as a new mother.

*“I think it has been up to us to seek that knowledge and then of course you get frustrated when you try to look for something about infant's and risk and such and then you cannot find it.”* [Woman no. 7]

Also related to health literacy, in addition to not seeking advice etc., the women believed that their worries were too insignificant to request help from health visitors and general practitioners even though they frequently experienced worries. The women described not wanting to call the health visitor for questions about their infant’s social well-being during times with the COVID-19 pandemic because they did not want to bother them. Moreover, other women found it difficult to articulate their worries about adaption to motherhood and the infant’s needs via the telephone and instead they gave up on seeking advice from healthcare providers in advance. Nonetheless, the women described that they used to “slip in” worries they thought were perhaps too insignificant during the health visitor’s “normal” home visits; e.g. talks about routines and motherhood insecurity.

*“You feel discharged from the healthcare system. Both from the health visitors and the guidance. I am well aware that I am always welcome to contact a health visitor if there is something wrong, but I don’t have a very specific thing I need answers to. It's more these good discussions about topics we have during our meetings” [Woman no. 3]*

Moreover, most of the women found it frustrating that information during times with COVID-19 was only focused on numbers of identified cases and deaths and not on social and mental health during the lockdown. They missed an emphasis on how to structure everyday life throughout the new reality of COVID-19.

## Discussion

Our findings provide in-depth insights into the experiences of women with recent GDM during the COVID-19 lockdown. The lockdown had a negative impact on everyday life e.g. routines, loneliness and worries for the infant’s social well-being; but some also reported better family dynamics. The women were generally not worried about the risk of becoming sick in relation to COVID-19, but focused on the possible consequences from this e.g. breastfeeding difficulties and impact on everyday routines. Engaging in healthy behaviours was considered challenging by many of the women and they were worried about their risk for T2DM and GDM in future pregnancies. The women missed peer support from mothers’ groups and face-to-face visits from health visitors and found it difficult to navigate the restructured care with online and telephone set-ups.

### Worries and difficulties during the COVID-19 lockdown

The worries perceived during the lockdown were largely similar to those described elsewhere during pre-COVID-19 times among women with recent GDM and women with an infant in general, e.g. tiredness, everyday work, social and family life and the infant’s needs [32, 33]. However, our results indicate that these worries seem to have been intensified during the lockdown. Furthermore, we found that feelings of loneliness were described as more pronounced during the lockdown; this has been confirmed by other studies investigating mental health among women during times with COVID-19 [15, 20, 34]. Taken together, these findings warrant attention to mental health in women, including those with recent GDM, during COVID-19, and to support a focus on motherhood adaptation and the infant’s social progression as common worries in the infant period.

### Health behaviours during the lockdown

Health behaviour changes after delivery have the potential to reduce the risk of T2DM among women with prior GDM [35]. We found that healthy behaviours were adversely impacted among many of the women during lockdown; however, some found more time to prioritise being physically active during everyday life. Prior research conducted during pre-COVID-19 times among women with recent GDM identified similar barriers for health behaviour changes as those identified in our study; tiredness, lack of time and competing duties [32]. Yet, we found that it was more difficult for the women to engage in healthy behaviours because additional barriers were introduced during the lock-down. These enhanced barriers could influence weight gain and result in a less physically active everyday life, which have been reported to be adversely influenced during COVID-19 amongst the general population [36]. Considering our participants and their increased risk of T2DM such findings are particularly important for women with recent GDM.

### Healthcare access

The women in our study described how they refrained from contacting healthcare providers during the first COVID-19 lockdown. This is in line with an observed decline in patient requests in general practice in Denmark during the first months of lockdown [37]. However, our findings suggest a potential discrepancy between the women’s actual need for support and the support delivered to them, i.e. the restructured health visitor support and the telephone/online set-up. This is a shortfall that commands attention.

The restructured health visitor care during the Danish lockdown starting 11 March 2020 changed how the support was delivered to Danish families with an infant. A study by Karavadra et al. among pregnant women in the United Kingdom found the virtual set-ups during COVID-19 to provide “impersonal” care and the women as a result provided hereby less information to their healthcare providers; especially about mental health concerns [17].

Our findings confirm that women had difficulty navigating the restructured health visitor support with online/telephone contacts. We found that a barrier for contacting healthcare providers was that the women found it difficult to describe their worries without face-to-face contact, especially when these worries concerned motherhood adaptation, the infant’s social progression and everyday routines. Similarly, a study from the United Kingdom found that the restricted face-to-face healthcare professional support during the lockdown affected postpartum women negatively and that support were not sufficiently bridged virtually [21]. Taken together, these results indicate that health literacy among the women may become challenged when care is restructured as was the case during the lockdown. This fits with the overall understanding of health literacy, which is defined not only as a combination of personal competencies but also as situational resources and the healthcare systems’ complexity and accessibility [28]. Hence, the healthcare systems’ complexity and level of accessibility influence health literacy levels in populations, i.e. the population’s interaction with healthcare providers [38].

Our results may indicate that the restructured care consequently to COVID-19 introduced a *non*-health literacy responsive healthcare system not taking into account the women’s health literacy needs, e.g. difficulties in describing needs and worries without face-to-face contact. This underlines the importance of taking into account health literacy discrepancies, i.e. the variable ability to access healthcare services and to describe individual needs when supporting women with recent GDM during COVID-19.

### Worries about diabetes risk and COVID-19

Interestingly, we found that the women in our study were not particularly worried about the risk of becoming sick with COVID-19. This may be explained by the high level of trust towards the social and healthcare system among Danish citizens [39]. Nevertheless, the women were worried about potential consequences of contracting coronavirus, especially the impact on their ability to breastfeed. Our findings about breastfeeding uncertainties is in line with findings from “The COVID-19 New Mum study” from UK that found a feeling of too little support for breastfeeding among women during times with lockdowns [14]. Given the important benefits of breastfeeding for the growth and metabolic health of infants and mothers with GDM [40, 41], ensuring support and information for women with recent GDM about how to deal with COVID-19 and breastfeeding simultaneous therefore seems highly relevant. This indicates altogether a need for more focus on supporting breastfeeding during times with lockdowns, among others by accessible information on potential difficulties of breastfeeding in relation to coronavirus.

Finally, the women reported more intense worries about T2DM and re-occurrence of GDM in future pregnancies and they felt left alone without support for addressing these risks during the lockdown. A qualitative study found similar feelings of abandonment and insecurity about how to address T2DM risk among women with recent GDM in pre-COVID-19 times [42]. However, the lack of access to healthcare and peer support may have enhanced negative feelings about T2DM and GDM risk during lockdown. Thus, the importance of sustaining support may be an important lesson for future COVID-19 lockdowns.

### Strengths and limitations

By using a qualitative design we provided an in-depth understanding of the experiences and challenges faced by women with recent GDM during COVID-19. We collected much of our data by telephone, which may be a deficient alternative to face-to-face interviews since it limits ability to follow up on visual cues [43]. However, we addressed this limitation by offering the participants a comfortable set-up, i.e. time to finish their explanations and follow-up on sound cues during the interview. The interviewer’s experience in interacting with women with recent GDM has likely also been a positive factor for facilitating a safe environment.

We applied credibility, dependability and transferability as our validity criteria [29]. We approached credibility by presenting representative quotations from our data. Moreover, we sought agreement on the data analysis among all co-authors by peer triangulation [31], which was important to ensure we remained open-minded as themes emerged from the data.

Regarding dependability [29], it is important to note that COVID-19 has been progressing diversely over time in Denmark, i.e. lockdown restrictions have changed. This progression is a contextual factor which will likely impact individual experience of the lockdown among women. However, we sought to ensure dependability by asking all participants the same open-ended questions despite contextual changes in the data collection period. Moreover, interviewing is an evolving process during which interviewer and interviewee gain new insights into the phenomenon being studied [25]. However, the interview focus might have changed as the COVID-19 situation changed along with our study timeline. Therefore, the results from this study could likely have been different if interviews were performed at another time with stricter or fewer restrictions and increased knowledge about COVID-19. However, we believe this study adds important knowledge about how the COVID-19 situation has been experienced among women and the potential effects it might have had for these.

Regarding transferability [29], our study was conducted only among Danish women. Importantly, our participants were recruited from a research study focusing on preventing T2DM [26] and the participants may therefore be more aware of T2DM risk and the effects of health behaviour changes compared to other women with recent GDM and likely more aware of health behaviours than women with infants in the general population. Unfortunately, we were only able to recruit women who were living with a partner. Even though women with recent GDM is a potential group with higher needs for care after delivery, single parents are likely also a group with specific needs for support and potentially more vulnerable to social isolation etc. during COVID-19.

### Implications for research and practice

Delivering support during COVID-19 and the lockdowns should focus on motherhood adaptation, everyday routines, mental health, breastfeeding insecurity and the infant’s social well-being. These recommendations are likely also relevant to other high risk groups of women, e.g. groups who have experienced complicated pregnancies and/or have risk of adverse health outcomes after delivery; and potentially also to women in the general population.

The restructured care with introduction of telephone/online set-ups seems to reduce the level of support provided to the women. There was a discrepancy between the women’s actual need for support and the support being delivered to them. We suggest that face-to face care should be highly prioritised during COVID-19, if possible, because it facilitates a positive setting for the women to share worries and seek advice, which is limited during telephone/online set-ups. In case of telephone/online interactions, healthcare providers should initiate a dialogue about the common (yet intensified) worries during the first year after delivery and strive for establishing trustful relations where these worries can be addressed.

Moreover, health literacy difficulties are critical to incorporate when organising care during COVID-19. Hence, healthcare providers should emphasise to the women that they should reach out to their health visitor and general practitioner when perceiving any concerns. This count for any concerns including the infant’s social well-being, motherhood adaptation and mental health.

## Conclusions

The COVID-19 lockdown affected various aspects of everyday life such as routines, loneliness and worries for the infant’s social well-being among Danish women with recent GDM. They also found it difficult to navigate the restructured care. The online/telephone set-ups seemed to inhibit the women’s ability to address their worries for adaptation to motherhood and the infant’s social well-being. Healthy behaviours were considered difficult to achieve during lockdown, resulting in experience of intensified worries for T2DM and GDM risk. Moreover, the women expressed concerns about potential breastfeeding difficulties in case of coronavirus infection not being addressed in the care during the lockdown. Our results indicate a need for the healthcare system to be additionally accessible and responsive to health literacy difficulties, such as continuation in the use of the healthcare services and to address common motherhood worries in the first year after delivery.

## Data Availability

This study uses qualitative data and the participants did not consent to having the full transcripts of the interviews made publicly available. Making these available would furthermore hamper participant privacy. The data generated and analysed during the current study are therefore not publicly available due to individual privacy among the participants in this study. Please contact NHJ in case of any questions regarding the data used for this study.

## Abbreviations

GDM: Gestationel diabetes mellitus
T2DM: Type 2 diabetes mellitus

## References

[1] Organization WH (2020). COVID 19 Public Health Emergency of International Concern (PHEIC). Global research and innovation forum: towards a research roadmap. Edited by Preparedness GRCfID.

[2] Caramelo F, Ferreira N, Oliveiros B. Estimation of risk factors for COVID-19 mortality - preliminary results. medRxiv. 2020 2020.2002.2024.20027268.

[3] Haj Bloukh S, Edis Z, Shaikh AA, Pathan HM (2020). A look behind the scenes at COVID-19: National Strategies of infection control and their impact on mortality. Int J Environ Res Public Health.

[4] World Health Organization Coronavirus Disease (COVID-19) Dashboard [https://covid19.who.int/].

[5] Whiting DR, Guariguata L, Weil C, Shaw J (2011). IDF diabetes atlas: global estimates of the prevalence of diabetes for 2011 and 2030. Diabetes Res Clin Pract.

[6] Fødte og Fødsler 1997- [Births and Deliverys 1997-] [https://www.esundhed.dk/Registre/Det-medicinske-foedselsregister/Foedte-og-foedsler-1997-og-frem].

[7] Metzger BE, Lowe LP, Dyer AR, Trimble ER, Chaovarindr U, Coustan DR, Hadden DR, McCance DR, Hod M, McIntyre HD (2008). Hyperglycemia and adverse pregnancy outcomes. N Engl J Med.

[8] Vounzoulaki E, Khunti K, Abner SC, Tan BK, Davies MJ, Gillies CL (2020). Progression to type 2 diabetes in women with a known history of gestational diabetes: systematic review and meta-analysis. BMJ.

[9] Song C, Lyu Y, Li C, Liu P, Li J, Ma RC, Yang X (2018). Long-term risk of diabetes in women at varying durations after gestational diabetes: a systematic review and meta-analysis with more than 2 million women. Obes Rev.

[10] Clausen TD, Mathiesen ER, Hansen T, Pedersen O, Jensen DM, Lauenborg J, Damm P (2008). High prevalence of type 2 diabetes and pre-diabetes in adult offspring of women with gestational diabetes mellitus or type 1 diabetes. Role Intrauterine Hyperglycemia.

[11] Hinkle SN, Buck Louis GM, Rawal S, Zhu Y, Albert PS, Zhang C (2016). A longitudinal study of depression and gestational diabetes in pregnancy and the postpartum period. Diabetologia.

[12] Ruohomäki A, Toffol E, Upadhyaya S, Keski-Nisula L, Pekkanen J, Lampi J, Voutilainen S, Tuomainen TP, Heinonen S, Kumpulainen K (2018). The association between gestational diabetes mellitus and postpartum depressive symptomatology: a prospective cohort study. J Affect Disord.

[13] Kragelund Nielsen K, Groth Grunnet L, Terkildsen Maindal H, Workshop tDDA, Speakers W (2018). Prevention of type 2 diabetes after gestational diabetes directed at the family context: a narrative review from the Danish diabetes academy symposium. Diabet Med.

[14] Vazquez-Vazquez A, Dib S, Rougeaux E, Wells JC, Fewtrell MS (2021). The impact of the Covid-19 lockdown on the experiences and feeding practices of new mothers in the UK: preliminary data from the COVID-19 new mum study. Appetite.

[15] Dib S, Rougeaux E, Vázquez-Vázquez A, Wells JCK, Fewtrell M (2020). Maternal mental health and coping during the COVID-19 lockdown in the UK: data from the COVID-19 new mum study. Int J Gynecol Obstet.

[16] Overbeck G, Graungaard AH, Rasmussen IS, Andersen JH, Ertmann RK, Kragstrup J, Wilson P. Pregnant women's concerns and antenatal care during COVID-19 lock-down of the Danish society. Dan Med J. 2020;67(12):1–7. 33269695

[17] Karavadra B, Stockl A, Prosser-Snelling E, Simpson P, Morris E (2020). Women’s perceptions of COVID-19 and their healthcare experiences: a qualitative thematic analysis of a national survey of pregnant women in the United Kingdom. BMC Pregnancy Childbirth.

[18] Shorey SY, Ng ED, Chee CYI (2021). Anxiety and depressive symptoms of women in the perinatal period during the COVID-19 pandemic: a systematic review and meta-analysis. Scand J Public Health.

[19] Barbosa-Leiker C, Smith CL, Crespi EJ, Brooks O, Burduli E, Ranjo S, Carty CL, Hebert LE, Waters SF, Gartstein MA (2021). Stressors, coping, and resources needed during the COVID-19 pandemic in a sample of perinatal women. BMC Pregnancy Childbirth.

[20] Provenzi L, Grumi S, Altieri L, Bensi G, Bertazzoli E, Biasucci G, Cavallini A, Decembrino L, Falcone R, Freddi A, Gardella B, Giacchero R, Giorda R, Grossi E, Guerini P, Magnani ML, Martelli P, Motta M, Nacinovich R, Pantaleo D, Pisoni C, Prefumo F, Riva L, Scelsa B, Spartà MV, Spinillo A, Vergani P, Orcesi S, Borgatti R, MOM-COPE Study Group. Prenatal maternal stress during the COVID-19 pandemic and infant regulatory capacity at 3 months: A longitudinal study. Dev Psychopathol. 2021:1–9. 10.1017/S0954579421000766. Epub ahead of print. 10.1017/S0954579421000766 34210369

[21] Jackson L, De Pascalis L, Harrold JA, Fallon V, Silverio SA. Postpartum women's experiences of social and healthcare professional support during the COVID-19 pandemic: a recurrent cross-sectional thematic analysis. Women Birth. 2021. 10.1016/j.wombi.2021.10.002 PMC8553649 34756734

[22] Miller T (2007). “Is this what motherhood is all about?”: weaving experiences and discourse through transition to first-time motherhood. Gend Soc.

[23] Aoyagi S-S, Tsuchiya KJ (2019). Does maternal postpartum depression affect children's developmental outcomes?. J Obstet Gynaecol Res.

[24] Irwin JL, Davis EP, Hobel CJ, Coussons-Read M, Dunkel Schetter C (2020). Maternal prenatal anxiety trajectories and infant developmental outcomes in one-year-old offspring. Infant Behav Dev.

[25] Brinkmann S, Kvale S. Doing Interviews Second edn. London: SAGE; 2018.

[26] Nielsen KK, Dahl-Petersen IK, Jensen DM, Ovesen P, Damm P, Jensen NH, Thogersen M, Timm A, Hillersdal L, Kampmann U (2020). Protocol for a randomised controlled trial of a co-produced, complex, health promotion intervention for women with prior gestational diabetes and their families: the face-it study. Trials.

[27] Francis JJ, Johnston M, Robertson C, Glidewell L, Entwistle V, Eccles MP, Grimshaw JM (2010). What is an adequate sample size? Operationalising data saturation for theory-based interview studies. Psychol Health.

[28] IUHPE (2018). IUHPE position statement on health literacy: a practical vision for a health literate world. Glob Health Promot.

[29] Graneheim UH, Lundman B (2004). Qualitative content analysis in nursing research: concepts, procedures and measures to achieve trustworthiness. Nurse Educ Today.

[30] Graneheim UH, Lindgren B-M, Lundman B (2017). Methodological challenges in qualitative content analysis: a discussion paper. Nurse Educ Today.

[31] Denzin NK, Lincoln YS, Giardina MD (2006). Disciplining qualitative research. Int J Qual Stud Educ.

[32] Lie MLS, Hayes L, Lewis-Barned NJ, May C, White M, Bell R (2013). Preventing type 2 diabetes after gestational diabetes: women's experiences and implications for diabetes prevention interventions. Diabet Med.

[33] Ayers S, Crawley R, Webb R, Button S, Thornton A, group HAc (2019). What are women stressed about after birth?. Birth.

[34] Chrzan-Dętkoś M, Walczak-Kozłowska T, Lipowska M (2021). The need for additional mental health support for women in the postpartum period in the times of epidemic crisis. BMC Pregnancy Childbirth.

[35] Tuomilehto J, Lindström J, Eriksson JG, Valle TT, Hämäläinen H, Ilanne-Parikka P, Keinänen-Kiukaanniemi S, Laakso M, Louheranta A, Rastas M (2001). Prevention of type 2 diabetes mellitus by changes in lifestyle among subjects with impaired glucose tolerance. N Engl J Med.

[36] Flanagan EW, Beyl RA, Fearnbach SN, Altazan AD, Martin CK, Redman LM (2021). The impact of COVID-19 stay-at-home orders on health behaviors in adults. Obesity (Silver Spring, Md).

[37] The Danish Health Authority. COVID-19: Monitorering af aktivitet i sundhedsvæsenet [COVID-19: Monitoring of healthcare service activities]. Sundhedsstyrelsen [The Danish Health Authority]. Copenhagen; 2020. p. 1–39.

[38] Brach C, Keller D, Hernandez LM, Baur C, Parker R, Dreyer B, Schyve P, Kemerise AJ, Schillinger D (2012). Ten attributes of health literate health care organizations. Edited by Academies IoMotN.

[39] Böhm R, Lilleholt L, Zettler I, group CD. Denmark COVID-19 snapshot MOnitoring (COSMO Denmark): Monitoring knowledge, risk perceptions, preventive behaviours, and public trust in the current coronavirus outbreak in Denmark. PsychArchives. 2020.

[40] Gunderson EP (2014). Impact of breastfeeding on maternal metabolism: implications for women with gestational diabetes. Curr Diab Rep.

[41] Gunderson EP, Greenspan LC, Faith MS, Hurston SR, Quesenberry CP, Investigators SOS (2018). Breastfeeding and growth during infancy among offspring of mothers with gestational diabetes mellitus: a prospective cohort study. Pediatr Obes.

[42] Parsons J, Sparrow K, Ismail K, Hunt K, Rogers H, Forbes A (2018). Experiences of gestational diabetes and gestational diabetes care: a focus group and interview study. BMC Pregnancy Childbirth.

[43] Novick G (2008). Is there a bias against telephone interviews in qualitative research?. Res Nurs Health.

